# Response Inhibition in Autistic Adults: A Functional Near‐Infrared Spectroscopy Study in Virtual Reality

**DOI:** 10.1002/brb3.71249

**Published:** 2026-02-16

**Authors:** Anna Vorreuther, Nektaria Tagalidou, Katharina Lingelbach, Armin Hubert, Laura Bareiß, Tanja Nittel, Marc Ristau, Mathias Vukelić

**Affiliations:** ^1^ Institute of Human Factors and Technology Management IAT University of Stuttgart Stuttgart Germany; ^2^ Applied Neurocognitive Systems, Fraunhofer Institute For Industrial Engineering IAO Stuttgart Germany; ^3^ Applied Neurocognitive Psychology Carl Von Ossietzky University Oldenburg Germany; ^4^ auticon Deutschland GmbH Berlin Germany; ^5^ Institute of Educational Science, Department of Teaching and Learning with Intelligent Systems University of Stuttgart Stuttgart Germany

**Keywords:** autism, functional near‐infrared spectroscopy, inhibition, virtual reality

## Abstract

**Introduction:**

Response inhibition, a core component of executive functioning, has been studied extensively in autism, though results depend substantially on task choice and design. This study investigated whether autistic and non‐autistic adults differ in behavioral and neurophysiological responses during a visuospatial go/no‐go task (GNGT) implemented in virtual reality (VR).

**Methods:**

Participants (22 autistic, 10 non‐autistic) completed a blocked go/no‐go task in a VR environment, where stimuli appeared in varied spatial locations. Prefrontal hemodynamic responses were recorded using functional near‐infrared spectroscopy (fNIRS), along with reaction times (RTs) and error rates.

**Results:**

Both groups demonstrated slower RTs and fewer errors in no‐go blocks compared to go blocks, with no significant group differences in behavioral performance. fNIRS analyses revealed significant right‐lateralized increases in oxygenated hemoglobin concentration in the dorsolateral prefrontal cortex (dlPFC) during no‐go blocks in non‐autistic adults only. Autistic adults showed no significant task‐related modulation of prefrontal cortex activity.

**Discussion:**

While behavioral performance was comparable across groups, only non‐autistic participants showed task‐related modulation of dlPFC activity. These findings highlight differential neural engagement during inhibition and illustrate the potential of fNIRS paradigms for examining the executive functioning of autistic individuals in VR.

## Graphical abstract

In a VR‐based go/no‐go task combined with fNIRS, autistic and non‐autistic adults showed comparable behavioral performance, but only non‐autistic adults exhibited increased right dorsolateral prefrontal cortex activation during response inhibition, highlighting differential neural engagement and illustrating the potential of fNIRS paradigms for examining executive functioning of autistic individuals in VR.

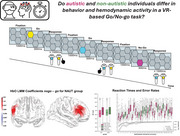

**Abbreviations**
ADHD: attention deficit/hyperactivity disorder
ANOVA: analysis of variance
ASC: autism spectrum condition
ASD: autism spectrum disorder
AUT: autistic group
BA: Brodmann area
CAVE: cave automatic virtual environment
CI: confidence interval
CLES: common language effect size
dlPFC: dorsolateral prefrontal cortex
fMRI: functional magnetic resonance imaging
fNIRS: functional near‐infrared spectroscopy
fOLD: fNIRS Optodes' Location Decider
GLM: general linear models
GLMM: generalized linear mixed model
GNGT: go/nogo‐task
HbO: oxygenated hemoglobin
HbR: deoxygenated hemoglobin
LED: light‐emitting diodes
LMM: linear mixed models
MEG: magnetoencephalography
NAUT: non‐autistic group
RT: reaction time
vlPFC: ventrolateral prefrontal cortex
VR: virtual reality
ΔRT: difference between RTs per block type

## 1 Introduction

Autism spectrum condition (ASC), or autism spectrum disorder (ASD), is a neurodevelopmental variation associated with difficulties in social interaction, as well as hyper‐ or hypo‐reactivity to sensory input (American Psychiatric Association 2013). Repetitive or restricted patterns of behaviors, interests, or activities may be observed in autistic individuals, though symptom expression varies strongly between individuals, as emphasized by the characterization as a spectrum (American Psychiatric Association 2013). One theoretical model for explaining the behavior of autistic individuals is a neurodivergence in executive functioning and associated neurophysiological processes compared to non‐autistic individuals (Demetriou et al. 2019; Hill 2004; St John et al. 2022). Within this framework, cognitive inhibition, which is the ability to cancel or suppress an action at will, is considered a factor potentially altered in autism (Demetriou et al. 2019; Diamond 2013; St John et al. 2022). While ASC is generally associated with deficits in cognitive functioning compared to neurotypical controls (Demetriou et al. 2019), a recent review reported that half of the reviewed studies on cognitive inhibition reported poorer performance of autistic adults relative to non‐autistic controls, while the remainder observed no significant differences or mixed results (St John et al. 2022).

The inconsistency in findings may be partly attributable to divergent operational definitions of cognitive inhibition and to methodological variability across studies. For instance, one study found that autistic adults performed better in a task if it had inherent structure, even though the task itself was complex (Kleinhans, Akshoomoff, and Delis 2005). There is a wide variability of paradigms, ranging from traditional neuropsychological experiments to ecologically valid tasks meant to assess inhibition skills needed in the “real world” (e.g., the Hayling Sentence Completion Task; Burgess and Shallice 1997). The latter tasks usually involve verbal semantic fluency, other cognitive functions, or spatial working memory skills, which may moderate the results (Demetriou et al. 2019; St John et al. 2022). In contrast, experimental paradigms specifically designed to target response inhibition, such as stop‐signal and go/no‐go tasks (GNGTs; Donders 1969), offer more controlled approaches to studying inhibitory processes.

GNGTs assess prepotent response inhibition (Geurts et al. 2014) by creating a need for action cancellation through response selection (Raud et al. 2020). In a typical GNGT, participants respond to “go” stimuli and refrain from responding to “no‐go” stimuli, allowing comparison of behavioral and neural markers of inhibition across blocks containing only go trials (i.e., “go” blocks) and blocks containing a mixture of go and no‐go trials (i.e., “no‐go” blocks; Herrmann et al. 2005; Hudak et al. 2017; Mayer et al. 2015; Raud et al. 2020). Typically, reaction times (RTs) in response to stimuli requiring a response, that is, go‐stimuli, would be overall faster in experimental blocks where no inhibition stimuli, that is, no‐go‐stimuli, are presented compared to experimental blocks where participants react to both (Donders 1969; Gomez et al. 2007; Miller and Low 2001).

Prior studies employing GNGTs report behavioral deficits in autistic adults (Lai et al., 2012; Raymaekers et al. 2004; Uzefovsky et al. 2016; Wilson et al. 2014), as well as no behavioral differences between groups (Langen et al. 2012; Prat et al. 2016; Schmitz et al. 2006; Shafritz et al. 2015; Yuk et al. 2020). Nevertheless, evidence from neuroimaging studies suggests distinctly different neural patterns associated with GNGT response inhibition (Duerden et al. 2013; Langen et al. 2012; Prat et al. 2016; Schmitz et al. 2006; Shafritz et al. 2015; Yuk et al. 2020). For example, in a GNGT using facial expression stimuli, no behavioral differences between groups were found, although functional magnetic resonance imaging (fMRI) measurements revealed differences in brain activation patterns during cognitive inhibition (Duerden et al. 2013). Specifically, both groups recruited various areas associated with the processing of facial expressions and the ventrolateral prefrontal cortex (vlPFC). However, the non‐autistic group, in contrast to the autistic group, additionally recruited the dorsolateral prefrontal cortex (dlPFC), an area often associated with response inhibition (Duerden et al. 2013; Li et al. 2023; Oldrati et al. 2016; Wessel and Anderson 2023).

In addition to studies employing stationary imaging methods like fMRI and magnetoencephalography (MEG), functional near‐infrared spectroscopy (fNIRS) presents a non‐invasive optical modality for measuring concentration changes of cortical oxygenated hemoglobin (HbO) and deoxygenated hemoglobin (HbR). These concentration changes are indicative of the current metabolic demands of the cortical regions of the brain (Ferrari and Quaresima 2012). Given its relative robustness to motion and higher spatial resolution compared to other mobile imaging methods, which allows for the localization of brain regions involved in cognitive inhibition, fNIRS is a promising mobile alternative to fMRI for investigating the brain in more applied experimental settings like Virtual reality (VR) paradigms (Lingelbach et al. 2023; Lühmann et al. 2021; Peck et al. 2014; Pinti et al. 2020; Quaresima and Ferrari 2019).

Prior research using fNIRS studies on response inhibition in autistic adults assessed through GNGT is limited (for review see Zhang and Roeyers 2019). We found one study employing the mobile method while assessing response inhibition in autistic adults with a stop‐signal task (Ishii‐Takahashi et al. 2014). They reported reduced HbO activation changes in vlPFC, albeit results from a stop‐signal task are not necessarily transferable to a GNGT (Littman and Takács 2017; Raud et al. 2020). Notably, fNIRS has been used numerous times for assessing response inhibition in autistic children (Prins et al. 2013; van der Oord et al. 2014; Yerys et al. 2013). Given the emerging understanding of the moderating roles of task design and participant variability (Demetriou et al. 2019; Littman and Takács 2017; Raud et al. 2020; St John et al. 2022) in response inhibition research on autistic adults, the assessment in more complex environments than standard laboratory settings warrants further exploration.

Traditional executive function tasks often lack ecological validity, potentially obscuring how autistic individuals engage with real‐world demands. VR allows researchers to simulate more complex and stimulus‐rich scenarios while retaining experimental control, making it a promising tool for studying cognitive processes in more dynamic environments. VR has emerged as a pivotal technology in the field of learning, offering immersive and engaging learning experiences (Herrera et al. 2008; Prins et al. 2013; van der Oord et al., 2014). It enables users to explore potential actions and visualize the consequences securely and cost‐effectively. At the same time, task settings (e.g., content, speed, and format) can be customized to individual preferences and skills (Rizzo and Galen 1997; Philippe et al. 2020).

Within autism research, VR has recently begun to be integrated with fNIRS. For example, a protocol was developed for the joint use of fNIRS and VR to study response inhibition in autistic children (Kuo et al. 2025). In addition, a recent proof‐of‐principle study assessed the feasibility of combining fNIRS measures with a cave automatic virtual environment (CAVE) and compared GNGT performances of autistic adults and children across CAVE‐based and screen‐based conditions (Dina et al. 2025). To date, however, no study (to our knowledge) has examined the feasibility of combining mobile, head‐mounted VR with fNIRS to assess response inhibition in autistic adults relative to non‐autistic controls.

In the present study, we adapted the well‐established GNGT to a VR environment. Our objective was to investigate cognitive inhibition in autistic and non‐autistic adults using a VR‐based GNGT, and to examine whether established behavioral and neurophysiological patterns observed in traditional settings could be replicated in this immersive environment. To investigate the neural mechanisms underlying task performance in these immersive settings, we employed fNIRS. Additionally, we measured RTs and error rates during the task. By combining behavioral and neurophysiological assessments in a VR‐based paradigm, this study seeks to bridge the gap between basic research on executive functions and contribute to the development of more personalized approaches to cognitive assessment and support tools in autistic adults.

## 2 Materials and Methods

### 2.1 Participants

A total of 44 participants were recruited for the study from six different testing sites across Germany. All spoke German as their native language. Sixteen participants of the total were allocated to the non‐autistic group and 28 participants to the autistic group. However, a total of 12 participants were excluded from the analysis, leaving a sample size of 32 participants. Three subjects were excluded due to an insufficient number of completed blocks. For two subjects, we encountered technical issues. For four subjects, experimental data were acquired with two separately calibrated recordings, hence making meaningful analysis of fNIRS data difficult. Another three participants were excluded during analysis due to insufficient fNIRS signal quality in the majority of channels. Thus, 22 autistic (x̅_age_ = 34.45 ± 8.94, ranging from 18 to 52; 18 right‐handed, 4 left‐handed; 15 male, 7 female) and 10 non‐autistic participants (x̅_age_ = 32.9 ± 6.69, ranging from 22 to 44; 10 right‐handed; 4 male, 6 female) were included in all analyses. The study was approved by the ethics commission of the University of Stuttgart (Germany; approval number 22‐009) and conducted according to the Declaration of Helsinki. All participants gave written informed consent prior to the experimental session.

Inclusion criteria for both autistic and non‐autistic participants included being 18–55 years of age, normal or corrected‐to‐normal vision and hearing, and having adequate German language skills. These criteria were chosen to ensure that each participant was, in principle, able to perform the VR task. Participants were identified as belonging to the autistic group (AUT) if diagnosed with ASC by a clinician according to the DSM‐V criteria (American Psychiatric Association 2013). Participants not diagnosed with ASC were included in the non‐autistic group (NAUT) if not diagnosed with any psychological or neurological disorder according to the DSM‐V. Further, non‐autistic individuals were not included in the study if they indicated regular intake of psychopharmaceuticals or medication affecting the central nervous system. The non‐autistic control group received a monetary reward for participation (30€). The autistic participants were recruited from a company and received compensation in the form of paid working hours.

We collected various demographical data prior to testing and assessed potential differences in groups using Welch's, Fisher's Exact, and Chi‐square tests depending on the nature of the tested variable (Fisher 1922; Pearson 1900; Welch 1947). Groups did not differ significantly on any of the demographic variables (see Table S1). In addition to collecting general demographical data, an fNIRS‐suitability score (Nagels‐Coune et al. 2020; Vorreuther et al. 2023) was determined for each participant to assess overall suitability for measurements with the light‐based modality regarding physical attributes like head size, hair, and skin tone (see Table S2). We collected this suitability score for group comparison to make sure that aforementioned features were not affecting signal acquisition significantly more often in one group than in the other. Groups did not differ significantly regarding NIRS suitability (AUT:  $\bar{x}=12.32\;\pm \;3.34$, ranging from 3 to 17; NAUT:  $\bar{x}=11.6\;\pm \;2.55$, ranging from 8 to 16; Welch's t(22.66) = ‐0.67, *p* = 0.51).

### 2.2 VR Headset and fNIRS Acquisition

**FIGURE 1:**
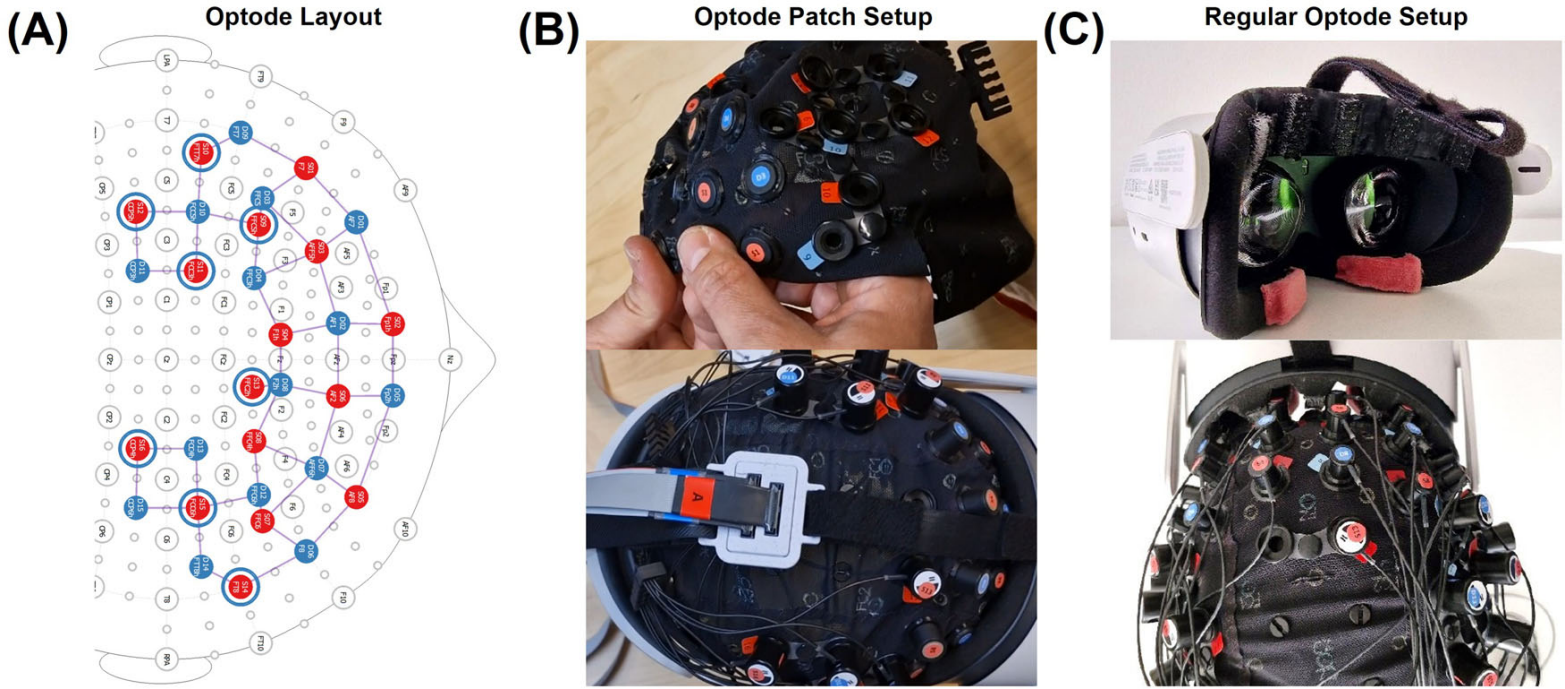
Optode setup. (A) 2‐d layout of fNIRS optode setup with light‐emitting sources (red) and light detectors (blue). Long‐distance channels are indicated by lilac lines connecting sources and detectors, and short‐distance channels are indicated by blue circles around corresponding sources. Note that the short‐distance channel of source S14 was not used, although shown in the layout, due to the VR headset applying pressure at this location. (B) Pictures of the optode patch setup with frontal flat optodes to fit under a VR headset. (C) Pictures of the setup with regular frontal optodes and cut‐out in VR‐headset foam.

The VR setup utilized the Meta Quest 2 headset as the primary hardware to present the GNGT. To see the participants’ field of view during the sessions, the SideQuest platform was used to connect the headset to a screen. The Quest 2 headset was modified for the specific needs of the experiment; the original head strap was adjusted in length, ensuring proper sensor placement while maintaining stability and user comfort during the VR experience. The data were recorded using the NIRSport2 system (NIRx Medizintechnik GmbH, Berlin, Germany), which employs dual light‐emitting diodes (LEDs) emitting light at two wavelengths (760 and 850 nm). The Aurora fNIRS recording software (NIRx Medizintechnik GmbH, Berlin, Germany) was used at a sampling rate of 5.8 Hz. The montage with 16 sources and 15 detectors (41 long‐ and 8 short‐distance channels totaling 52 channels) was determined using the fNIRS Optodes' Location Decider (fOLD; Zimeo Morais et al. 2018; see Figure 1A) to optimally investigate the brain regions of interest, namely the lateral prefrontal cortex roughly dividable into the dlPFC (Brodmann areas [BAs] 9 and 46) and vlPFC (BAs 44, 45, and 47). Naming conventions were derived from the Juelich brain atlas (version 3.1, Amunts et al. 2023; Amunts et al. 2020) and Brodmann (1909). Seven short distance channel were equally distributed across the montage to measure systemic physiological changes (Saager and Berger 2005; Santosa et al. 2020; Yücel et al. 2021). For one half of the sample, a newly developed optode patch with flattened optodes was used since it allowed unobtrusive measurements with the VR headset (see Figure 1B). However, due to a technical issue, the other half of the participants were measured with standard NIRSport 2 optodes. To enable unobtrusive measurements for the NIRSport 2 optodes, cut‐outs in the foam cover of the headset were made to fit the frontally placed optodes (see Figure 1C).

### 2.3 Experimental Setup

To execute the paradigm in the VR environment while simultaneously measuring fNIRS, experimental triggers, behavioral, and neurophysiological data were synchronized via Lab Streaming Layer (https://github.com/sccn/labstreaminglayer; accessed on November 23, 2023). The task and environment were built in Unity (version 2022.3.10f1; Unity Technologies 2023), and the VR headset sent triggers wirelessly via a local network to an acquisition computer. In addition to recording hemodynamic activation changes with the Aurora software, a custom‐built interface for the experimental supervisor was used to acquire behavioral data recorded by the controllers and headset, respectively.

### 2.4 Task

**FIGURE 2:**
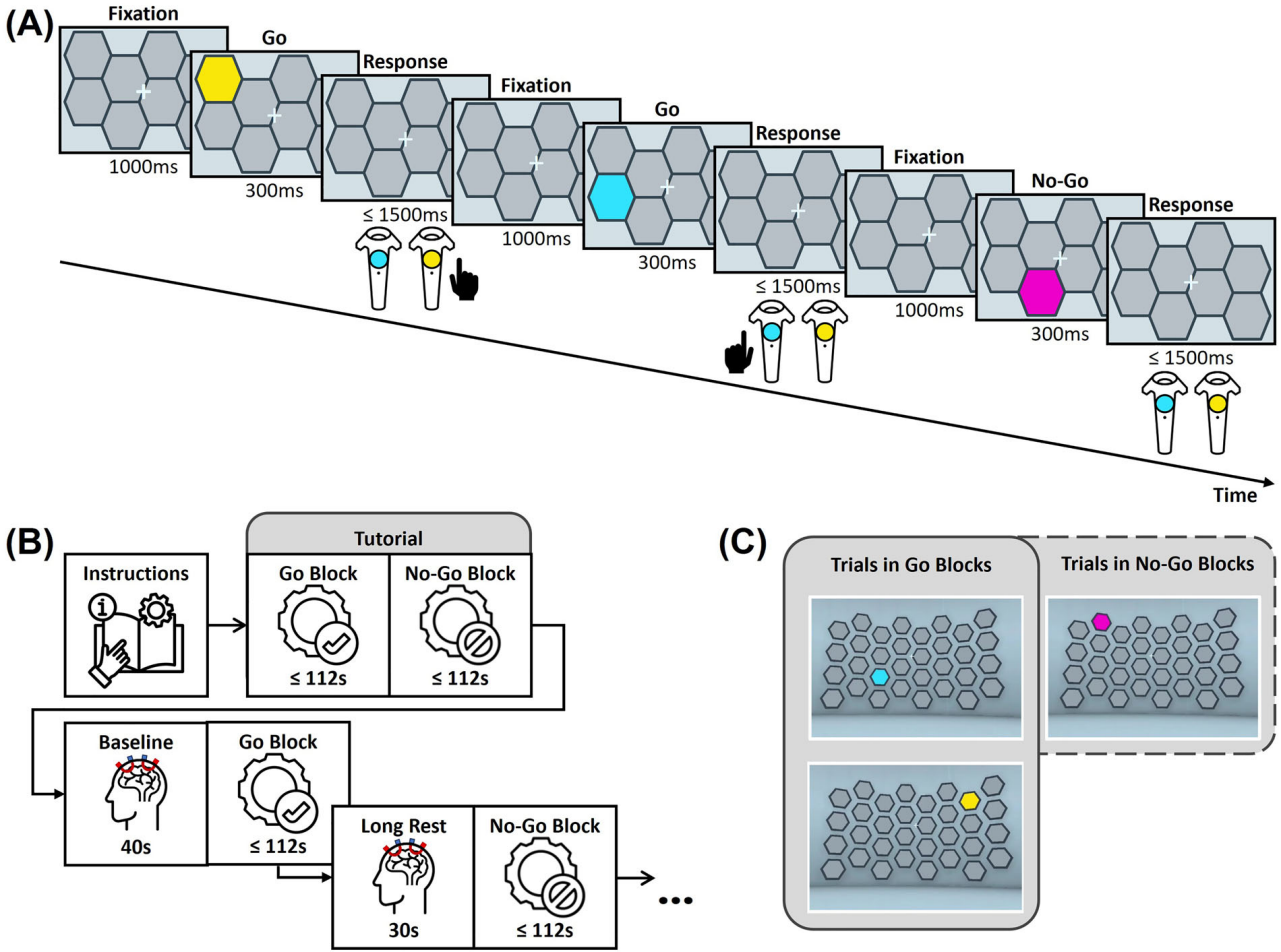
VR go/no‐go task. (A) Exemplary timeline of trials in a no‐go block. Each trial commenced with a resting phase of 1000 ms followed by stimulus presentation in the form of a color‐changing tile for 300 ms. Possible color changes were “go right” (yellow), “go left” (blue), or “no‐go” (magenta). The stimulus presentation was followed by a response time window of a maximum duration of 1500 ms. (B) Illustration of experimental procedure. Each experimental session started with task instructions and tutorial blocks of both condition types. Before the initial experimental block, a baseline recording of 40 s was completed for each participant. Between blocks, additional resting periods of 30 s were introduced. Note that the order of go and no‐go blocks was randomized. (C) Possible trial types per block type are illustrated with screenshots of the VR implementation of the paradigm during stimulus presentation.

In the present study, the task consisted of a GNGT, which was presented in a block design (Inoue et al. 2012). The implemented task rules were to press a button with either the left or right hand, depending on whether one of a set of grey tiles would change color to either blue or yellow, and to not press any button when the color changed to magenta. The number of grey tiles and thus possible locations for stimulus presentation was 32 (see Figure 2C).

### 2.5 Procedure

First, participants were introduced to the VR environment, and the headset was adjusted to fit their eye distance if necessary. Participants were then seated on a chair and prepared for fNIRS acquisition. Any unnecessary light sources (e.g., electric lights, bright sunlight) were reduced as much as possible to minimize interference with the fNIRS signal acquisition. Once the setup of the measurement equipment and VR hardware was completed, participants were asked to rate their perceived pain regarding the setup on a Wong‐Baker pain scale (Wong and Baker 1988; see Figure S1), which assesses pain on a scale from 0 (“no pain”) to 10 (“worst possible pain”) with the help of facial expressions. The rating was repeated after half of the testing runs and after the testing session was completed to ensure that participants felt comfortable wearing the combined fNIRS‐VR setup throughout the session.

At the beginning of each testing session, participants received instructions on how to perform the task in the VR environment and completed two training runs, one go block and one no‐go block. Training runs did not differ from testing runs other than the participants being aware that they were meant for training the task. To ensure that participants understood the rules, the experimental supervisor monitored the responses of the participants during training and testing runs. A 40‐s baseline resting‐state measurement was completed between the last training and the first testing run (see Figure 2B).

Each subsequent run consisted of a 30‐s baseline at the beginning, followed by 40 trials of the GNGT game. In total, each participant completed 16 runs, resulting in 640 trials in total. Runs were presented in a random order. Half of the runs consisted of only go trials, and the other half included 50% of no‐go trials as well, resulting in either a go block (only go trials) or a no‐go block (50% no‐go trials). The overall ratio of go and no‐go trials was 3:1. The number of presented no‐go trials was therefore 160 in total, and the number of go trials was 480. Each trial consisted of a fixation time window of 1000 ms, followed by stimulus presentation for 300 ms (a colored tile being presented), and a response window of time for the participant to respond to the stimulus (no longer than 1500 ms) before the next trial commenced (see Figure 2A).

### 2.6 Analysis

#### 2.6.1 Behavioral and Subjective Data Analysis

For the statistical analysis of go‐trial RTs, trials with RTs below 150 ms (Kosinski 2008; Ulrich and Miller 1994) or above 1000 ms (Ratcliff, 1993; Whelan, 2008) were excluded. This was based on the assumption that responses that were too fast or too slow indicated accidental button presses or a lack of attention.For the statistical analysis of go‐trial RTs, trials with RTs below 150 ms (Kosinski 2008; Ulrich and Miller 1994) or above 1000 ms (Ratcliff, 1993; Whelan, 2008) were excluded. This was based on the assumption that responses that were too fast or too slow indicated accidental button presses or a lack of attention.. This resulted in 2.29% of trials being excluded from behavioral analysis. Further, trials in which the responses were incorrect (4.3%) were excluded for RT analysis. Correct trials were then analyzed in a 2 (group) × 2 (block type) factors design with one between‐subjects factor group (AUT, NAUT) and one within‐subject factor block type (go blocks, no‐go blocks). RTs were log‐transformed after trial exclusions and before analysis to ensure a symmetrical distribution of the data for assessing distributional assumptions. As the assumptions of normality (Shapiro‐Wilk: W=0.996,p<0.001) and homogeneous variances (Levene's test: F(115,485)=2007.22,p<0.001) were not met for the log‐transformed RT data, non‐parametric statistical tests were applied. Raw RTs were used for the non‐parametric testing to preserve real‐world interpretability. A non‐parametric two‐sided Mann–Whitney *U*‐test for between‐subjects factor group (Mann and Whitney 1947) and Wilcoxon signed‐rank test for within‐subjects factor block type (Wilcoxon 1992) were computed alongside rank‐biserial correlations (Kerby 2014). Effect sizes were reported based on a brute‐force version of the common language effect size (CLES; Vargha et al. 2000; McGraw and Wong 1992). To test for an interaction effect, the difference between RTs per block type (ΔRT) was compared using a Mann–Whitney *U*‐test (Mann and Whitney 1947). A corresponding bootstrapped 95% confidence interval (CI; 10,000 iterations) was computed for all tests.

Error rates for both go‐ and no‐go trials were computed, namely omission errors (i.e., response was required yet incorrectly omitted), commission errors (i.e., response inhibition was required during a no‐go trial yet incorrectly a response was committed), and mistakes (i.e., a required yet incorrect response was committed). Again, trials considered implausible regarding a very fast RT were excluded from statistical analyses. To test for main and interaction effects between the independent variables on error rate, a generalized linear mixed model (GLMM) with a binomial distribution and logit link was fitted for all errors combined to increase statistical power. Effect sizes were reported using odds ratios with associated 95% CIs.

A mixed analysis of variance (ANOVA) with a 3 (time points) × 2 (group) factorial design was computed to evaluate the pain scores of the Wong–Baker scale. If the assumption of sphericity was violated (assessed through Mauchly's test), the Greenhouse–Geisser correction was applied and reported. Significance thresholds were set to *α *< 0.05 for all analyses. Effect sizes were reported using partial eta squared *η^2^_p_*. Post hoc paired‐samples *t*‐tests were used to test for differences between time points.

#### 2.6.2 Neurophysiological FNIRS Data Analysis

We preprocessed the fNIRS signals using MNE‐Python (version 1.6.1; Gramfort et al. 2014) and MNE‐NIRS (version 0.6.0; Luke et al. 2021) toolboxes. Preprocessing steps and decisions were chosen and reported in line with Yücel et al. (2021). First, we converted the raw data into an optical density measure. Next, channels with poor quality were excluded using the scalp coupling index as a quality measure with a threshold below 0.5 (Pollonini et al. 2014). To account for baseline shifts and spike artifacts, we applied a temporal derivative distribution repair (Fishburn et al. 2019). To correct systemic physiological interference, a short channel regression was used (Scholkmann et al. 2014). Short‐separation channels (i.e., channels with a distance below <10 mm) are mostly sensitive to blood perfusion and oxygenation changes in the extracerebral tissue layer (Scholkmann et al. 2014). The optical density data were transformed into HbO and HbR concentration changes with the modified Beer–Lambert Law and a partial pathlength factor of 6 (Gramfort et al. 2014; Huppert et al. 2009; Luke et al. 2021). Afterward, chromophore signals were filtered using a fourth‐order zero‐phase Butterworth bandpass filter with cutoff frequencies of 0.01 and 0.5 Hz and a transition bandwidth of 0.02 and 0.2 Hz.

For each experimental condition, we estimated the hemodynamic response participant‐wise using first‐level general linear models (GLM) with a canonical statistical parametric map hemodynamic response function. The GLM length of the time window was determined participant‐wise by the condition block with the shortest length. Additional regressors in the first‐level GLM included the HbO and HbR short channel signals, a third‐order polynomial drift, and an active baseline (Lingelbach et al. 2023; Yücel et al. 2021). Estimates for the contrast *no‐go—go* were obtained participant‐wise using the GLM coefficients (see Figure S3).

The second‐level coefficients per channel for each chromophore (HbO and HbR) and contrast were estimated using linear mixed models (LMM; Baayen et al. 2008; as implemented in pymer4, version 0.6.0) and the participant‐wise z‐standardized first‐level GLM coefficients. Participants were included as random intercepts in the models to account for non‐systematic interindividual differences. The LMM was estimated separately for the AUT and NAUT. To determine significant channels, we performed bootstrapping with 5000 iterations to calculate the 2.5th and 97.5th CI of the estimates (Cumming and Finch 2005) with a channel‐based Bonferroni correction (Lingelbach et al. 2023). Significant second‐level coefficients were projected onto a 3‐dimensional average brain template from both the rostral and lateral perspectives. To investigate group differences in brain activity between AUT and NAUT, hemodynamic responses in regions of interest identified by the LMM were compared between groups. Initially, first‐order theta estimates for each condition were baseline‐corrected. Next, grand averages and their 2.5th and 97.5th CI were estimated via bootstrapping (5000 iterations). Group differences were considered significant if the confidence intervals did not overlap (Cumming and Finch 2005).

## 3 Results

### 3.1 Behavioral and Subjective Results

**FIGURE 3:**
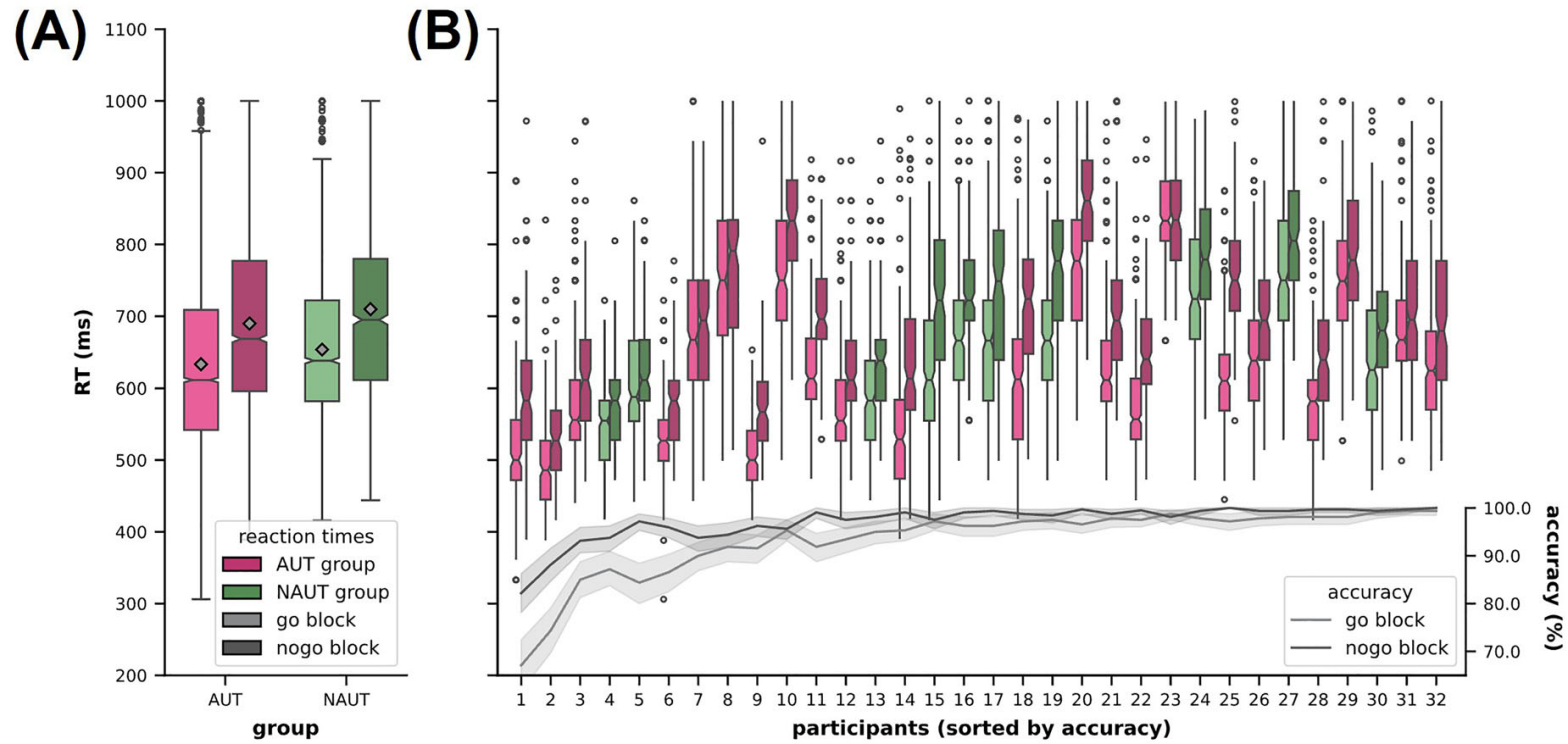
Reaction times and error rates. (A) The group averages of RTs (in ms) of go trials during go and no‐go blocks are visualized in box plots. Means are additionally indicated as round gray markers. (B) Individual RTs are plotted for each participant for each block type (go block: lighter colors; no‐go block: darker colors). Mean trial accuracies per block type are plotted for all participants on a secondary axis. AUT, autistic group; NAUT, non‐autistic group; RT, reaction times.

The Mann–Whitney *U*‐test for the main effect of group was not significant, indicating no difference in RTs of correct trials between groups (U=86,p<0.34,r=−0.22,CLES=0.4, mean difference  =  −19.67, 95% CI [−74.51; 35.62]; see Figure 3). The Wilcoxon signed‐rank test indicated a significant effect of block type (W=0,p<0.001,r=1.00,CLES=0.69), with go blocks yielding lower RTs than no‐go blocks (mean difference = 58.15, 95% CI [16.77, 99.66]; see Figure 3 and Table S5). This result indicates a complete directional consistency across participants, such that RTs were universally lower in go blocks. The Mann–Whitney U‐test for ΔRT indicated no significant interaction between group and block type (U=101,p=0.73,r=−0.08,CLES=0.46, mean difference = −1.61, 95% CI [−22.1, 19.48]).

The GLMM fitted to evaluate effects on error rates demonstrated acceptable fit to the data (Akaike's Information Criteria = 6756.5). There was a significant main effect of block type, indicating that participants were less likely to commit an error during no‐go blocks compared to go blocks (*b* = −0.92, SE = 0.08, *z* = −10.93, *p* < 0.001, OR = 0.40, 95% CI [0.34, 0.47]). The main effect of group was not statistically significant (*b* = −0.16, SE = 0.41, *z* = −0.39, *p* = 0.695, OR = 0.85, 95% CI [0.38, 1.90]), indicating no reliable difference in overall error likelihood between the groups. No significant interaction between group and block type was found (*b* = −0.17, SE = 0.18, *z* = −0.95, *p* = 0.340, OR = 0.85, 95% CI [0.60, 1.19]; see Figure 3B and Tables S6 and S7).

The Wong–Baker pain scores revealed a significant increase in perceived pain throughout the three assessment time points during the session, albeit the absolute ratings remained in the lower half of the scale on average ($F;1,60)=39.06,p\;<\;0.001,\eta_{p}^{2}=0.57$ (see Figure S2). Post hoc paired‐samples *t*‐tests showed that the differences were significant between all timepoints (for details see Tables S3 and S4). Descriptively, perceived pain was rated higher in AUT overall compared with NAUT, although no significant differences between groups were found. There was no interaction between time points and groups.

### 3.2 Neurophysiological Results

**FIGURE 4:**
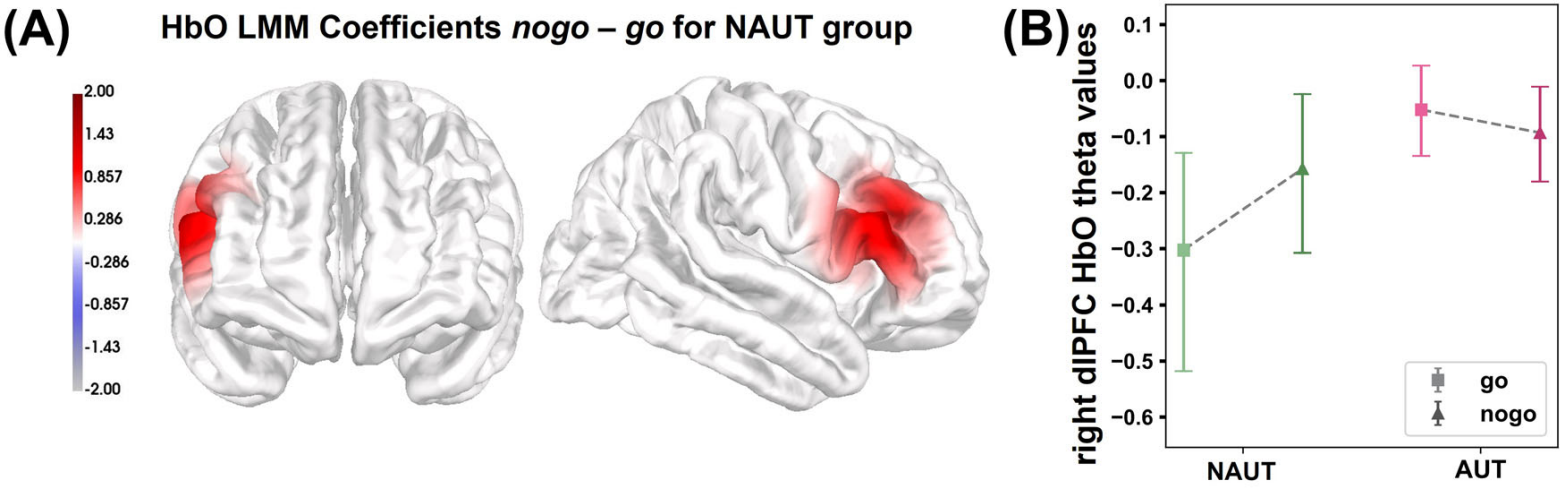
HbO LMM coefficients of the block type contrast and GLM estimates per group. (A) Significant second‐level linear mixed model (LMM) HbO estimates of the contrast *no‐go—go* projected onto a 3‐D brain surface viewed rostrally (left) and laterally (middle) for the non‐autistic group (NAUT). (B) Bootstrapped and baseline‐corrected mean first‐level GLM estimates (theta values) and confidence intervals (CIs) for each group per block type condition within the significant channel S7‐D12 corresponding to the right dlPFC. The channel was identified in the LMM for the NAUT. AUT, autistic group; dlPFC, dorsolateral prefrontal cortex; LMM, linear mixed model; HbR, deoxygenated hemoglobin; NAUT, non‐autistic group.

The second‐level LMM analysis revealed a significant effect of experimental condition only on HbO but not HbR concentration changes in the NAUT (see Figure 4A). The effects in the contrast *no‐go–go* were localized in the right hemisphere. Specifically, the bootstrap analysis of the first‐level GLM coefficients revealed one significant HbO channel, namely S7‐D12, for the NAUT. There was no significant effect of block type on HbO or HbR concentration changes for the AUT. The follow‐up region‐of‐interest analysis of the channel S7‐D12 revealed that the mean estimates varied more strongly between block conditions for the NAUT and were lower overall in the NAUT relative to the AUT (see Figure 4B and Table S8).

## 4 Discussion

We combined a VR‐based adaptation of a GNGT with fNIRS to investigate behavioral and neurophysiological differences between autistic and non‐autistic individuals during response inhibition. Our results demonstrate neural differences between groups and support the feasibility and sensitivity of using VR environments combined with fNIRS to investigate executive functioning in autistic and non‐autistic adults.

Behaviorally, both groups exhibited slower RTs and fewer errors during blocks requiring an inhibitory response (no‐go blocks compared to go blocks). The blocks including no‐go trials required the participants to inhibit their responses for half the stimuli, likely increasing conscious attention allocation to response selection, that is, increased time‐costly carefulness during the choice of response (see Figure 3). We observed no significant differences between groups in RTs and error rates. The absence of significant group differences in behavioral measures suggests that both groups engaged comparably with the task at the performance level. This aligns with prior findings that autistic adults can show similar executive task performance to non‐autistic adults, potentially reflecting the use of alternative cognitive strategies or compensatory mechanisms (Hill 2004; St John et al. 2022). Both groups made relatively few errors relative to the total number of trials (AUT: 5.26 ± 6.29%; NAUT: 3.48 ± 3.19%; see Figure 3 and Table S7), indicating that the task was not difficult overall.

Neurophysiologically, we observed right‐lateralized differences in HbO concentration changes in the NAUT between block‐type conditions. In the NAUT, HbO changes increased in a channel localized above the right dlPFC (BA 9 and 46; see Figure 4A). This finding is consistent with a broad body of research implicating the right dlPFC in cognitive inhibition and executive control (Friedman and Robbins 2022; Panikratova et al. 2020; Yuan and Raz 2014). Our result of increased HbO concentration changes in the right dlPFC during no‐go blocks met the hypothesis regarding the hemodynamic response associated with a GNGT in non‐autistic individuals. It corroborates previous findings implicating the right dlPFC in cognitive inhibition processes (Duerden et al. 2013; Li et al. 2023; Oldrati et al. 2016; Wessel and Anderson 2023).

In contrast, autistic participants exhibited no significant modulation of HbO concentration across conditions. This might imply the recruitment of the right dlPFC (as well as other measured areas) to an equal extent throughout the task, regardless of the presence of no‐go trials. Prior studies have highlighted that autistic individuals may rely on different neural architectures during executive function tasks (Duerden et al. 2013; Zhang and Roeyers 2019). The observed GLM estimates of fNIRS signal data recorded in the AUT were not significantly different and were closer to zero compared to the non‐autistic participants, especially relative to the estimate for no‐go blocks in the NAUT. While absolute HbO concentration changes alone cannot explain task performance, the pattern observed supports an association between dlPFC HbO modulation and inhibitory response control in NAUT, yet not in AUT. Autistic adults might have engaged in different task strategies, more efficient processing, or reduced reliance on canonical executive control networks.

### 4.1 Limitations

A few limitations should be considered when interpreting the results of our study. First, we did not extensively test for established correlating factors like intelligence quotient (Geurts et al. 2014) and psychological comorbidities, for example, attention deficit/hyperactivity disorder (ADHD, 38.5% comorbidity with ASC; Rong et al. 2021) associated with decreased inhibition abilities (Fried et al. 2016). Variability in the study population regarding autism is unavoidable, given the heterogeneity of autism itself. The diagnosis of ASC was the main inclusion criterion, and we aimed to exclude individuals solely based on the fNIRS measurement suitability. Thus, we only included a questionnaire on standard demographic data and an fNIRS suitability assessment. Within the scope of our tests, the non‐autistic and autistic groups did not differ significantly on demographic variables, including educational background, current employment, and age (see Table S1). Moreover, the unequal group sizes represent a limitation and may affect statistical power and the generalizability of the findings. Although we balanced recruitment efforts across groups, practical constraints resulted in a discrepancy in final sample sizes. Thus, further research with a more differentiated assessment of the sample would be ideal to advance research on cognitive inhibition in autistic adults.

Second, the overall low error rates across both groups are indicative of a potential ceiling effect. Although the distribution of errors was similar across groups (see Figure 3B), a more challenging task may have revealed more subtle behavioral differences. Future iterations could incorporate adaptive difficulty mechanisms to maintain an optimal level of challenge and better account for potential learning or habituation effects during the session (Enriquez‐Geppert et al. 2013). Considering that increased cognitive load can disproportionately impact autistic individuals (St John et al. 2022), this aspect warrants further systematic investigation.

Lastly, the patch prototype was designed to fit optodes under the VR headset and corresponding head strap, thereby allowing comfortable placement of both, as indicated by overall low pain assessment scores (see Figure S2). It should be noted that the optodes were therefore not placed in exact accordance with the international 10–20 EEG system layout to investigate frontal regions as proposed by the fOLD toolbox. While our results are still indicative of the underlying hemodynamics, the non‐standard layout makes replicability unnecessarily complicated. We included the montage files in Supporting Information to support replication and extension of our work. Moreover, the patch malfunctioned during data acquisition, and therefore an improvised second optode setup was used for the remainder of the acquisition (see Figure 1). This introduced a potential source of variability in participants that, although not leading to significant differences regarding the scalp‐coupling indices (see Table S9), should be acknowledged.

### 4.2 Implications and Future Directions

This study demonstrates that VR‐based cognitive paradigms combined with fNIRS can be effectively applied to investigate executive functioning in autistic adults. Our findings contribute to a more nuanced understanding of inhibitory processes in autism and validate the use of VR‐fNIRS integration for future research. Although behavioral performance was similar across groups, distinct neurophysiological patterns emerged, underscoring the need to examine individual differences in cognitive strategies and neural processing.

The combined use of an fNIRS cap and a VR headset can introduce physical discomfort and potential interference with optode placement. Although participants in the present study reported only mild discomfort on average, future research may further improve the ergonomics of such combined setups. Possible strategies include the use of lightweight or rear‐balanced VR headsets, low‐profile or flexible optode holders such as the presented patch setup, or custom adapter frames to reduce direct contact between the headset and the fNIRS cap. Session designs incorporating brief breaks and guidance to minimize unnecessary head movement are recommended to support participant comfort and signal stability. These refinements may enable more seamless integration of VR and fNIRS in future applied or clinical research.

These results also highlight the possibility of developing personalized support tools relying on VR environments, potentially exploring real‐time neurofeedback based on fNIRS signals. Gamified, home‐based systems that promote intrinsic motivation through personalized, immersive scenarios represent a promising direction (Keshavan et al. 2014; Lumsden et al. 2016). While similar tools already exist, primarily for children and typically based on behavioral performance alone (van der Oord et al. 2014; Prins et al. 2013; Hudak et al. 2017; for review see Karami et al. 2021), the integration of neurophysiological feedback could enhance their precision and adaptability. Prior work has shown that neurofeedback can facilitate learning and improve executive functions (Nouchi et al. 2021), and its combination with VR scenarios may amplify these benefits (Kober et al. 2016). However, a study specifically investigating the outcome of such a VR‐based neurofeedback tool should be conducted before drawing clinical or applied conclusions.

## 5 Conclusion

This study validated the integration of fNIRS with a VR‐based GNGT to investigate behavioral and neurophysiological differences between autistic and non‐autistic adults. While both groups showed comparable behavioral performance, only non‐autistic participants exhibited task‐related modulation of the right dlPFC. These findings replicate known inhibition‐related neural patterns of non‐autistic participants in an immersive setting and reveal differential neural engagement in autistic individuals. Future research should explore this neural heterogeneity further in ecologically valid and immersive contexts and may build on these results to explore the use of fNIRS‐informed VR tools tailored to autistic adults.

## Author Contributions

Conceptualization: Anna Vorreuther, Nektaria Tagalidou, Mathias Vukelić, and Laura Bareiß. Data curation: Anna Vorreuther, Nektaria Tagalidou, and Katharina Lingelbach. Formal analysis: Anna Vorreuther and Katharina Lingelbach. Funding acquisition: Mathias Vukelić. Investigation: Anna Vorreuther, Nektaria Tagalidou, Laura Bareiß, and Tanja Nittel. Methodology: Anna Vorreuther, Nektaria Tagalidou, and Armin Hubert. Project administration: Laura Bareiß, Tanja Nittel, and Marc Ristau. Resources: Mathias Vukelić. Software: Armin Hubert and Marc Ristau. Supervision: Mathias Vukelić. Validation: Anna Vorreuther, Nektaria Tagalidou, Katharina Lingelbach, and Mathias Vukelić. Visualization: Anna Vorreuther and Katharina Lingelbach. Writing – original draft: Anna Vorreuther, Nektaria Tagalidou, and Katharina Lingelbach. Writing – review and editing: Anna Vorreuther, Nektaria Tagalidou, Katharina Lingelbach, Armin Hubert, Laura Bareiß, Tanja Nittel, Marc Ristau, and Mathias Vukelić.

## Funding

This work was supported by grants from the German Federal Ministry for Education and Research (BMBF: 16SV8722).

## Conflicts of Interest

The authors declare no potential conflicts of interest concerning the research, authorship, and/or publication.

## Supporting information




**Supplementary Material**: brb371249‐sup‐0001‐SuppMat.pdf

**Supplementary Material**: brb371249‐sup‐0002‐SuppMat.zip

## Data Availability

Preregistration of this study can be found at https://osf.io/sr5y3. Code and data pertaining to the results presented will be made available upon request.
