# Supplementary material for: Response Inhibition in Autistic Adults: A Functional Near‐Infrared Spectroscopy Study in Virtual Reality
Source: Brain Behav. 2026 Feb 16;16(2):e71249. doi: 10.1002/brb3.71249 (PMC12909282; doi:10.1002/brb3.71249)
Supplement: Supplementary file 1 — Supplementary Material: brb371249‐sup‐0001‐SuppMat.pdf [file BRB3-16-e71249-s001.pdf]

Supplemental Material

Table 1

Demographics

Variable	AUT (n = 22)	NAUT (n = 13)	Test statistic	*p*
**age (years)**	34.45 ± 8.94 (18–52)	32.9 ± 6.69 (22–44)	Welch's t (23.04) = -0.55	0.59 (ns)
**sex**			Fisher's Exact Test, OR = 0.31	0.24 (ns)
female	7	6		
male	15	4		
**handedness**			Fisher's Exact Test, OR = ∞	0.28 (ns)
left	4	0		
right	18	10		
**education**			$\chi^2(5) = 4.90$	0.43 (ns)
basic education	1	0		
high school diploma	9	2		
vocational school	4	0		
bachelor's degree	5	5		
master's degree	2	3		
doctorate	1	0		
**employment**			$\chi^2(3) = 4.41$	0.22 (ns)
full-time employee	19	6		
full-time student	1	3		
part-time student	1	0		
unemployed	1	1		

**coffee / drug / medication intake**			Fisher's Exact Test, OR = 0.47	0.44 (ns)
yes	15	5		
no	7	5		
**head size (cm)**	57.89 ± 1.90 (53–61)	57.0 ± 2.05(53–60)	Welch's t (16.31) = -1.16	0.26 (ns)
**cap size**			Fisher's Exact Test, OR = ∞	0.31 (ns)
size 58	22	9		
size 60	0	1		
**glasses**			Fisher's Exact Test, OR = 0.38	0.64 (ns)
yes	5	1		
no	17	9		

Note. Note that coffee, drug, and medicine intake was assessed for 12 hours prior to the experimental session in one joint item and is thus reported here jointly. Abbreviations: AUT: autistic group; OR: odds ratio; ns: not significant; NAUT: non-autistic group

Table 2

FNIRS Suitability Score

Attribute	Description	Value
hair length	bald	0
	shaved	1
	short I (<3cm)	2
	short II (>3cm)	3
	long (>20cm)	4
hair color	bald	0
	light (blond, white)	1
	medium (dark blonde, light brown, red, grey)	2
	dark (dark brown, black)	3
hair (root) thickness	bald	0
	fine	1
	medium	2

	dense	3
hair density	bald	0
	thin	1
	medium	2
	thick	3
hair structure	bald	0
	straight	1
	wavy	2
	curly	3
skin	light	1
	tanned	2
	dark	3
biological sex	female	0
	male	1
head circumference	< 58 cm	0
	58 - 60 cm	1
	> 60 cm	2
score		$\sum$

Note. For each participant, one value per attribute was chosen based on the assigned description. The sum of all values determined the fNIRS suitability score, specifically a low score indicates better suitability for the light-based method.

Figure 1

Illustration of Wong-Baker Pain Scale

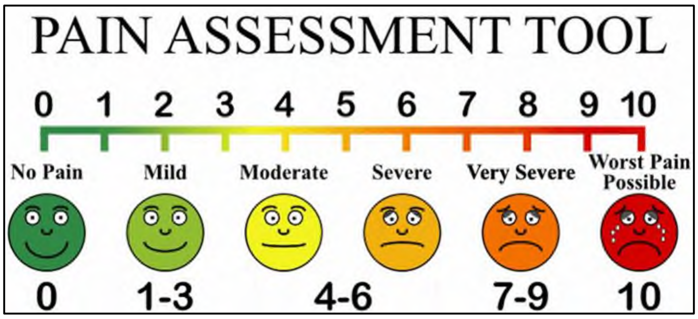

Note. The pain scale was printed out on an A4 paper and presented to the participants prior to equipping the VR-headset. During the experimental session, participants were asked to indicate verbally one number at the beginning, the middle, and the end of the experimental session.

Figure 2

Perceived Pain During the FNIRS Measurement

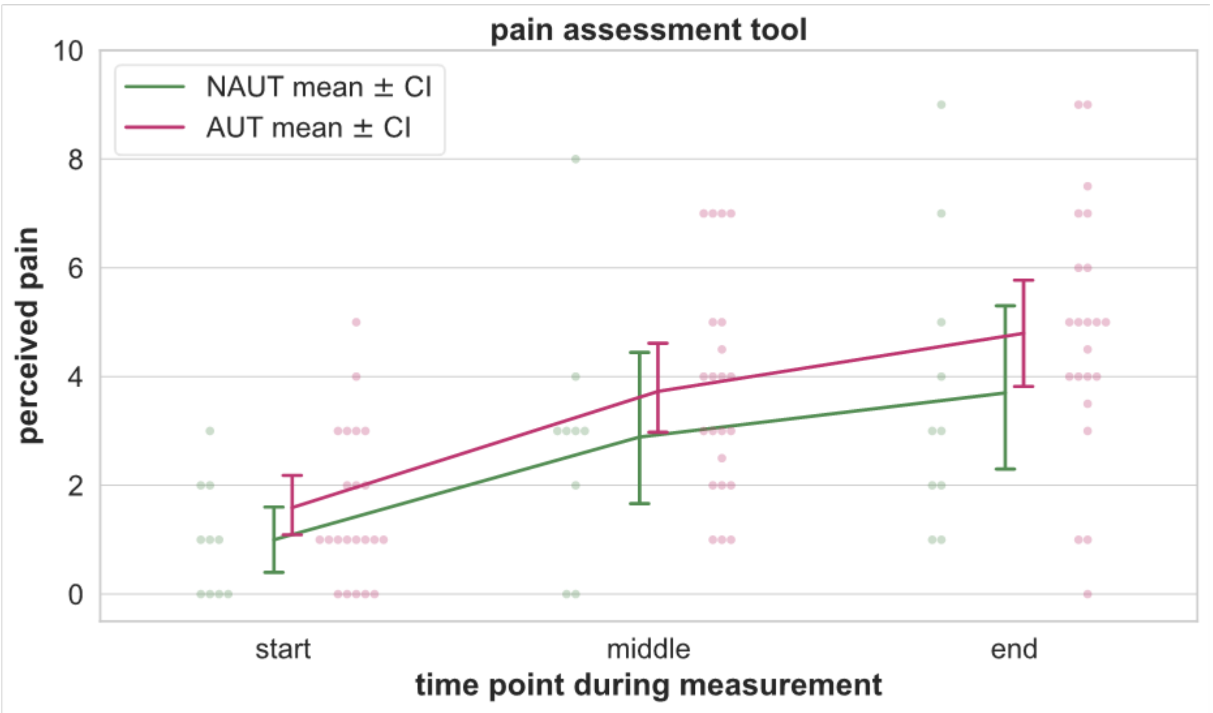

Note. Perceived pain as assessed by the Wong-Baker pain scale is plotted for each assessment time point during fNIRS measurement. Group means and 95% confidence intervals (CIs) are plotted as bars and individual scores of participants are depicted as scattered dots. Abbreviations: AUT: autistic group; NAUT: non-autistic group

Table 3

Mixed ANOVA for Pain Assessment Scores

Variable	SS	Df1	Df2	MS	F	p	η_p^2	ϵ
group	13.932	1	29	13.932	1.649	0.209	0.054	-
timepoint	144.038	2	58	72.019	39.057	0.000	0.574	0.901
interaction	1.847	2	58	0.924	0.501	0.609	0.017	-

Note. Abbreviations: df: degrees of freedom; MS: mean square; SS: sum of squares; η_p^2: effect size indicated as partial eta squared; ϵ: Greenhouse-Geisser epsilon factor of sphericity assumption

Table 4

Paired-Samples T-Tests for Pain Assessment Scores Over Timepoints

Timepoint A	Timepoint B	Mean ± STD (A)	Mean ± STD (B)	*W*	*p*	Hedges
end	middle	4.44 ± 2.48	3.48 ± 2.09	51	0.008	0.41
end	start	4.44 ± 2.48	1.45 ± 1.31	7.5	< 0.001	1.48
middle	start	3.48 ± 2.09	1.45 ± 1.31	0	< 0.001	1.15

Note. Abbreviations: STD: standard deviation

Table 5

Statistical Results for Reaction Times

Test (dv)	Statistic	*p*	*r*	CLES	Mean difference [bootstrapped 95% CI]	Comparison
Mann-Whitney (ΔRT)	*U* = 101	0.730	-.082	0.46	-1.608 [-22.095, 19.479]	interaction group × block type
Mann-Whitney (RT)	*U* = 86	<.339	-.218	0.39	-19.666 [-74.507, 35.622]	group
Wilcoxon Signed Rank (RT)	*W* = 0	<.001	1.0	0.69	58.147 [16.769, 99.664]	block type

Note. Abbreviations: CI: confidence interval; dv: dependent variable; r: rank biserial coefficient; RT: reaction times

Table 6

Generalized Linear Mixed Model Fixed Effects for Error Rates

Regression results	Coefficient [95%CI]	Standard error	z	*p*	Odds ratio [95%CI]
intercept	-3.065 [-3.514, -2.617]	0.229	-13.392	.000	0.047 [0.030, 0.073]
group (NAUT)	-0.160 [-0.962, 0.642]	0.409	-0.392	.695	0.852 [0.382, 1.900]
block type (no-go)	-0.916 [-1.081, -0.752]	0.084	-10.929	.000	0.400 [0.339, 0.471]
group × block type	-0.168 [-0.512, 0.177]	0.176	-0.954	.340	0.846 [0.599, 1.193]

Note. Abbreviations: CI: 95% confidence interval; NAUT: non-autistic group

Table 7

Participant-Wise Descriptive Statistics for Each Error Type by Group

Error type	Group	Mean (%) ± SD	SEM	Median (%)	IQR	Min (%)	Max (%)
all erroneous trials	AUT	5.26 ± 6.29	1.34	2.42	5.12	0.31	25.51
	NAUT	3.48 ± 3.19	1.01	2.27	1.68	0.78	9.53
commission error	AUT	0.36 ± 0.74	0.16	0.08	0.29	0.00	3.32
	NAUT	0.13 ± 0.18	0.06	0.00	0.27	0.00	0.48
mistake error	AUT	4.53 ± 5.45	1.16	2.02	4.13	0.31	21.52
	NAUT	2.95 ± 2.91	0.92	1.94	2.01	0.47	9.08
omission error	AUT	0.38 ± 0.59	0.13	0.00	0.59	0.00	2.19
	NAUT	0.39 ± 0.60	0.19	0.16	0.12	0.00	2.03

Note. This table reports participant-wise descriptive statistics for four types of response errors across the two groups AUT (N = 22) and NAUT (N = 10). Values represent the percentage of trials per participant on which a given error occurred. Abbreviations: AUT: autistic group; IQR = interquartile range; NAUT: non-autistic group; SEM = standard error of the mean.

Figure 3

Illustrative Example of the First-Level GLM Design Matrix

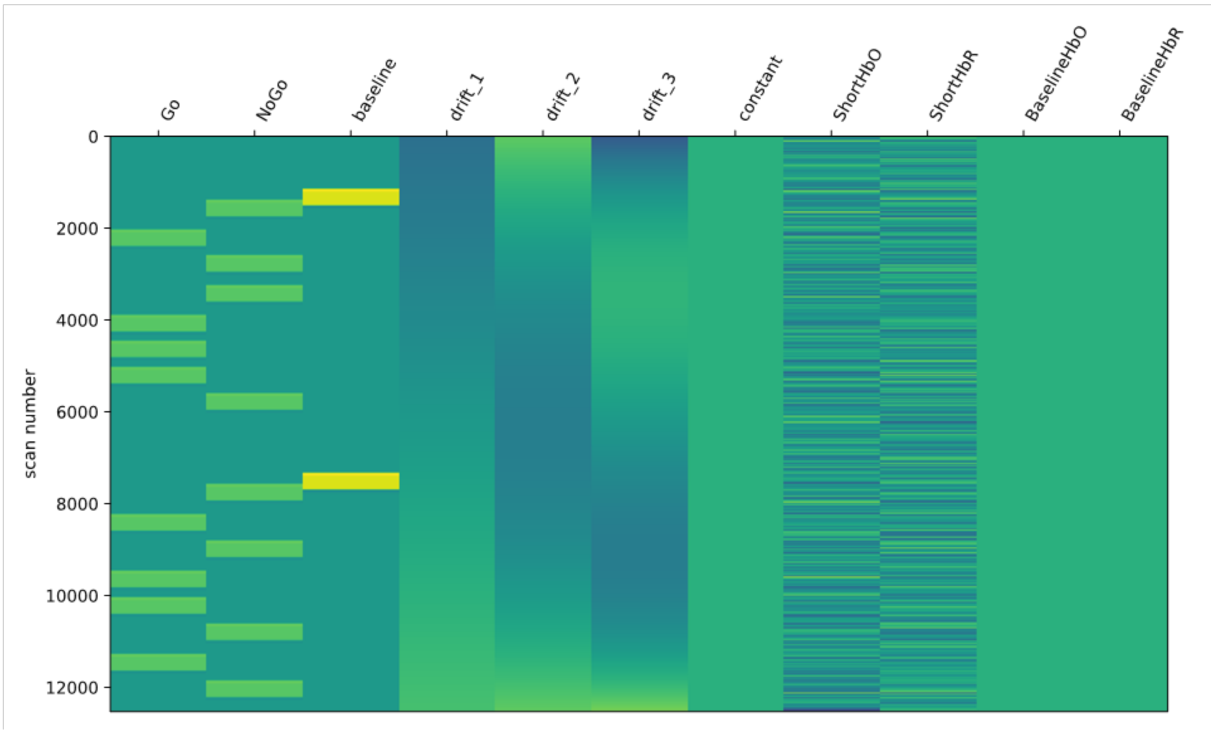

Note. Example of regressors in design matrix from one participant. Each column represents one regressor over scans (rows).

Table 8

First-Level General Linear Model Estimates of Channel S7-D12

Group – block type condition	Theta values (mean [95% CI])
NAUT – go	-0.302 [-0.518, -0.129]
NAUT – no-go	-0.158 [-0.307, -0.024]
AUT – go	-0.052 [-0.135, 0.027]
AUT – no-go	-0.093 [-0.18, -0.011]

Note. Mean and 95% confidence intervals of first-level GLM estimates (theta values) corresponding to channel S7-D12 for each block type condition (go, no-go) per group (AUT, NAUT). Abbreviations: AUT: autistic group; CI: confidence interval; GLM: General Linear Model; NAUT: non-autistic group

Table 9

Scalp Coupling Index (SCI) and Bad Channels per Optode Layout

Variable	Z	*p*	N_P	N_R	$Mean_P$ [+SD, -SD]	$Mean_R$ [+SD, -SD]
number of bad channels	1.73	0.07	10	22	9.03 [4.8, 14.4]	4.36 [2.82, 6.27]
mean SCI	-1.91	0.06	10	22	0.87 [0.81, 0.92]	0.93 [0.91, 0.95]
SD SCI	1.75	0.08	10	22	0.21 [0.17, 0.26]	0.17 [0.15, 0.19]

Note. To ensure that no significant variability was introduced by the varying optode setups, we compared the number of bad fNIRS channels and scalp coupling indices between the participants grouped by optode setups using a Mann-Whitney U-test. Channels were considered bad if the scalp-coupling-index was below 0.5. Abbreviation: P: patch setup; R: regular setup; SCI: scalp coupling index; SD: standard deviation
