# Supplementary figures and images for: Response Inhibition in Autistic Adults: A Functional Near‐Infrared Spectroscopy Study in Virtual Reality
Source: Brain Behav. 2026 Feb 16;16(2):e71249. doi: 10.1002/brb3.71249 (PMC12909282; doi:10.1002/brb3.71249)

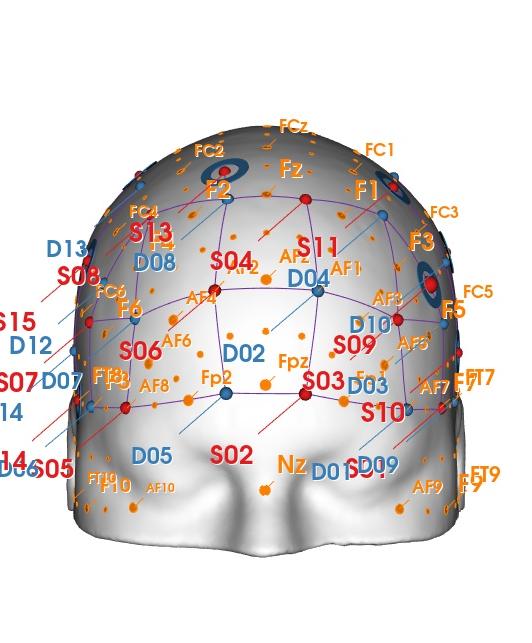

Supplement: Supplementary file 2 — Supplementary Material: brb371249‐sup‐0002‐SuppMat.zip [file BRB3-16-e71249-s002.zip › fNIRS_optode_montage/3d_head_views/anterior.jpeg]

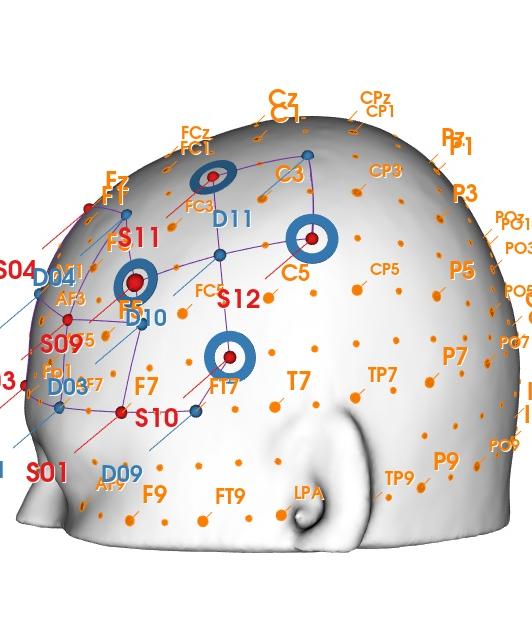

Supplement: Supplementary file 2 — Supplementary Material: brb371249‐sup‐0002‐SuppMat.zip [file BRB3-16-e71249-s002.zip › fNIRS_optode_montage/3d_head_views/left.jpeg]

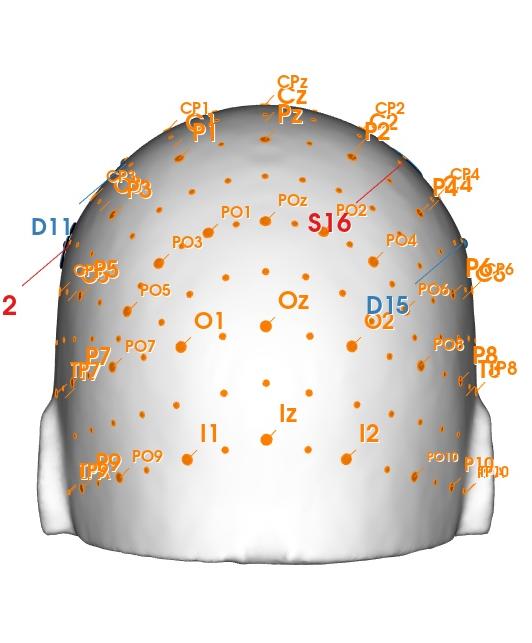

Supplement: Supplementary file 2 — Supplementary Material: brb371249‐sup‐0002‐SuppMat.zip [file BRB3-16-e71249-s002.zip › fNIRS_optode_montage/3d_head_views/posterior.jpeg]

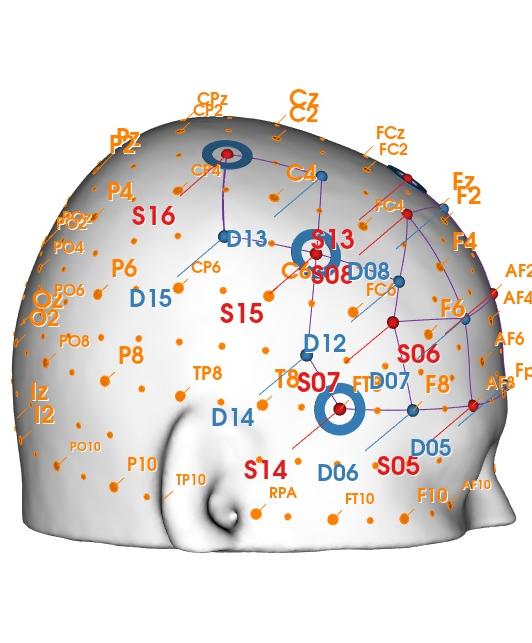

Supplement: Supplementary file 2 — Supplementary Material: brb371249‐sup‐0002‐SuppMat.zip [file BRB3-16-e71249-s002.zip › fNIRS_optode_montage/3d_head_views/right.jpeg]

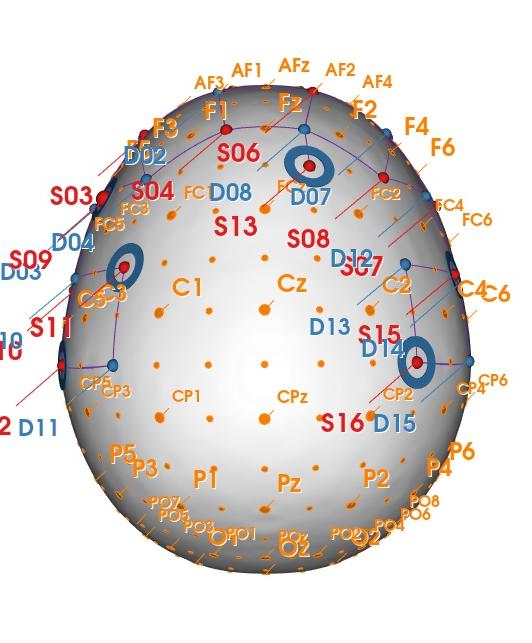

Supplement: Supplementary file 2 — Supplementary Material: brb371249‐sup‐0002‐SuppMat.zip [file BRB3-16-e71249-s002.zip › fNIRS_optode_montage/3d_head_views/top.jpeg]

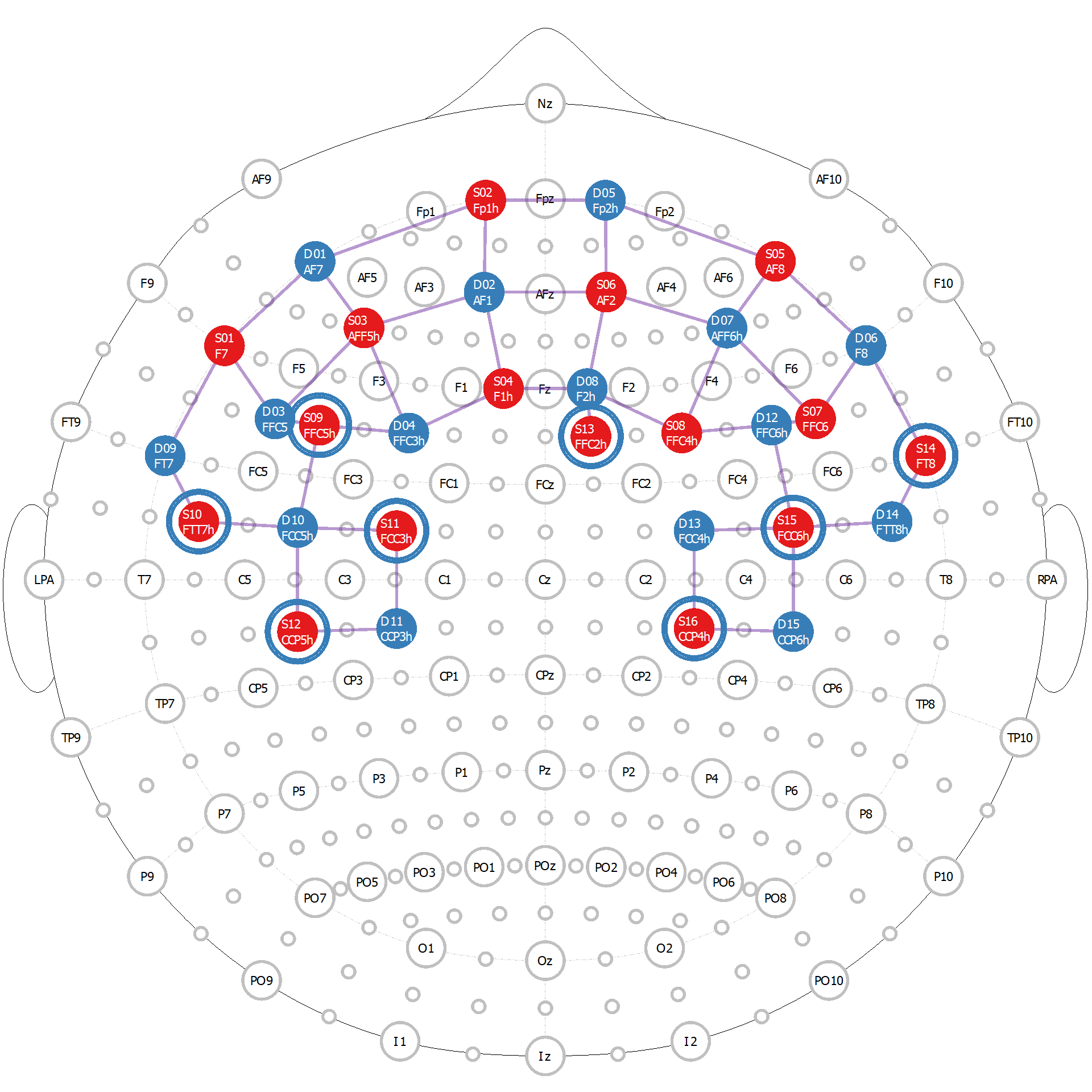

Supplement: Supplementary file 2 — Supplementary Material: brb371249‐sup‐0002‐SuppMat.zip [file BRB3-16-e71249-s002.zip › fNIRS_optode_montage/fNIRS_optode_montage.png]
